# An atrial-fibrillation-linked connexin40 mutant is retained in the endoplasmic reticulum and impairs the function of atrial gap-junction channels

**DOI:** 10.1242/dmm.013813

**Published:** 2014-03-13

**Authors:** Yiguo Sun, Xiaoling Tong, Honghong Chen, Tao Huang, Qing Shao, Weixiong Huang, Dale W. Laird, Donglin Bai

**Affiliations:** 1 Department of Physiology and Pharmacology, The University of Western Ontario, London, ON N6A 5C1, Canada.; 2 Anatomy and Cell Biology, The University of Western Ontario, London, ON N6A 5C1, Canada.

**Keywords:** Atrial fibrillation, Genetics, Gap junctions, Connexin40, Germline mutation

## Abstract

Connexin40 (Cx40)-containing gap-junction channels are expressed in the atrial myocardium and provide a low-resistance passage for rapid impulse propagation. A germline mutation in the *GJA5* gene, which encodes Cx40, resulting in a truncated Cx40 (Q49X) was identified in a large Chinese family with lone (idiopathic) atrial fibrillation (AF). This mutation co-segregated with seven AF probands in an autosomal-dominant way over generations. To test the hypothesis that this Cx40 mutant affects the distribution and function of atrial gap junctions, we studied the Q49X mutant in gap-junction-deficient HeLa and N2A cells. The Q49X mutant, unlike wild-type Cx40, was typically localized in the cytoplasm and failed to form gap-junction plaques at cell-cell interfaces. When the Q49X mutant was co-expressed with Cx40 or Cx43, the mutant substantially reduced the gap-junction plaque formation of Cx40 and Cx43. Electrophysiological studies revealed no electrical coupling of cell pairs expressing the mutant alone and a significant decrease in the coupling conductance when the mutant was co-expressed with Cx40 or Cx43. Further colocalization experiments with the organelle residential proteins indicate that Q49X was retained in the endoplasmic reticulum. These findings provide evidence that the Q49X mutant is capable of impairing gap-junction distribution and function of key atrial connexins, which might play a role in the predisposition to and onset of AF.

## INTRODUCTION

Atrial fibrillation (AF) is the most common cardiac arrhythmia and predisposes affected individuals to an increase in morbidity and mortality (Magnani et al., 2011). The majority of individuals with AF have underlying cardiovascular diseases, such as hypertension, heart failure, diabetes, valvular heart disease, myocardial ischemia or other ventricular dysfunction. However, some individuals develop AF without any known risk factors, known as lone (or idiopathic) AF (Lip et al., 2012). Accumulating studies suggest that there is a genetic predisposition for some AF cases (Brugada et al., 1997; Fox et al., 2004; Pfeufer et al., 2010). The first familial case of AF was reported in 1943 (Wolff, 1943) and the first genetic locus of AF was mapped to the 10q22–q24 site in 1997 (Brugada et al., 1997). In 2003, a putative genetic mutation linked to AF was identified in a gene encoding a potassium-channel subunit in a Chinese family (Chen et al., 2003). A clinical trial has also suggested that a parental history of AF in only one parent nearly doubles the AF risk for their children (Fox et al., 2004). By association, some genetic variants related to the mechanism of AF have been identified from individuals with AF, including genes encoding for potassium and sodium channels, gap-junction channel proteins and signaling molecules (Chen et al., 2003; Yang et al., 2004; Darbar et al., 2008; Hodgson-Zingman et al., 2008; Tsai et al., 2008; Xiao et al., 2011). Thus, channel and signaling molecules have become the focus in an attempt to reveal the pathophysiology of the inherited form of arrhythmia and to facilitate the understanding of the molecular mechanisms underlying AF.

Gap junctions are clusters of intercellular channels that couple neighbor cells electrically and metabolically, which is known as gap junctional intercellular communication (GJIC) (Giovannone et al., 2012). In the heart, gap junctions are responsible for rapid conduction of action potentials between cardiomyocytes. The gap-junction protein isoforms in human atria are Cx40, Cx43 and Cx45. Cx40 and Cx43 are expressed in working atrial myocytes, whereas Cx45 is primarily expressed in the sinus node (Gros et al., 1994; Davis et al., 1995). The co-existence of Cx40 and Cx43 in the atrial myocytes allows for possible connexin intermixing and the formation of homomeric, heteromeric, homotypic and heterotypic gap-junction channels (Valiunas et al., 2001; Cottrell et al., 2002). Previous studies using *Cx40* gene-knockout mice suggested that Cx40 is responsible for the decreased conduction velocity in the atrial myocardium and an increased susceptibility to inducible arrhythmias (Kirchhoff et al., 1998; Simon et al., 1998; Hagendorff et al., 1999). However, more recent studies indicated an increased conduction velocity and a decrease in the conduction heterogeneity was associated with the *Cx40* knockout mouse (Bagwe et al., 2005; Beauchamp et al., 2006; Leaf et al., 2008), questioning the roles of Cx40 in mouse atrial arrhythmias. In human clinical studies, both somatic and germline *GJA5* gene (encoding Cx40) mutations as well as other genetic variants in the regulatory regions of *GJA5* were found to associate with AF (Firouzi et al., 2004; Gollob et al., 2006; Juang et al., 2007; Yang et al., 2010b; Yang et al., 2010a; Wirka et al., 2011; Sun et al., 2013). This clinical outcome might be rooted in the function of Cx40, because our earlier studies indicate that some somatic and germline Cx40 missense mutants showed impaired gap-junction function (Gollob et al., 2006; Sun et al., 2013). A decrease in the abundance of Cx40 was also linked to AF (Firouzi et al., 2004; Wirka et al., 2011). The only germline truncation mutation of Cx40, Q49X, identified thus far was found in seven individuals belonging to a large Chinese family (Yang et al., 2010a). The Cx40 Q49X mutant co-segregated with AF in this Chinese family over generations and was not found in other unaffected family members or in 200 healthy individuals (Yang et al., 2010a).

**TRANSLATIONAL IMPACT**

**Clinical issue**

Atrial fibrillation (AF) is the most common form of cardiac arrhythmia (irregular heartbeat), affecting millions of individuals worldwide. AF increases the risks of morbidity and mortality and is a major cause of embolic stroke, because of a greater potential for blood clots to form in the heart. Genetic factors are known to play an important role in AF, and researchers have examined familial cases of AF to identify genetic mutations associated with the disease. Recently, analysis of a Chinese family with seven AF individuals led to the identification of a putative causative mutation in the *GJA5* gene, which encodes an atrial gap-junction protein, connexin40 (Cx40). The mutation, which co-segregated with the seven AF probands in an autosomal-dominant manner, results in a truncated form of Cx40. The functional basis for the association of truncated Cx40 with AF remains unknown.

**Results**

In this study, the authors sought to test the hypothesis that the AF-linked Cx40 mutant (Q49X) affects the distribution and function of atrial gap junctions. Using gap-junction-deficient HeLa and N2A cell lines, they demonstrate that the Q49X mutant is retained primarily within the endoplasmic reticulum (ER), rather than being localized to cell-cell junctions. In line with this, cells expressing the mutant protein failed to form gap-junction plaques at cell-cell interfaces. When the Q49X mutant was co-expressed with wild-type Cx40 or another major atrial gap-junction protein, Cx43, gap-junction-plaque formation was substantially reduced and the wild-type proteins were also sequestered in the ER, indicating a dominant-negative effect. Finally, electrophysiological analysis revealed a lack of electrical coupling of cell pairs expressing the mutant alone and a significant decrease in the coupling conductance when the mutant was co-expressed with Cx40 or Cx43.

**Implications and future directions**

These findings provide evidence that the Q49X mutant is unable to form functional gap junctions because it is retained intracellularly, in the ER. Furthermore, the Q49X mutant impairs gap-junction formation and coupling conductance driven by the two major atrial connexins. Thus, the truncated Cx40 mutant is likely to slow down heart impulse propagation in the atria and promote atrial arrhythmias. This study provides a molecular basis for the association of truncated Cx40 with AF. Understanding the mechanisms by which AF-linked Cx40 mutants impair gap-junction coupling could help in the development of effective treatments for this prevalent cardiac arrhythmia.

**Fig. 1:**
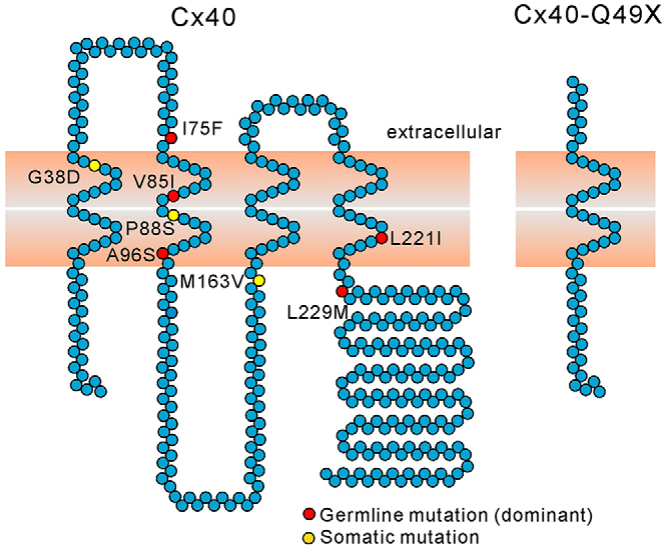
**Known lone-AF-linked Cx40 mutants.** Composite model of Cx40 denoting the locations of mutations associated with AF. AF-linked somatic (yellow circles) and germline (red circles) Cx40 mutations are illustrated. All of the AF-linked germline Cx40 mutants are autosomal-dominantly inherited. The Q49X mutant was predicted to be a loss-of-function mutant because it retains only the N-terminus, first transmembrane domain and a small part of the first extracellular domain.

In the present study, we characterized the Q49X AF-linked mutant, which consists of only the N-terminus, first transmembrane domain and a small part of the first extracellular loop of Cx40 (Fig. 1). Our results indicate that the Q49X mutant was not localized to cell-cell junctions but was retained within the endoplasmic reticulum (ER). Interestingly, the Q49X mutant also displayed dominant-negative effects on the formation of functional gap junctions composed of Cx40 or Cx43. These findings in mammalian cell lines are consistent with the idea that the truncated Cx40 mutant could impair the overall gap-junction function in the atria and possibly play a role in the pathogenesis of AF.

## RESULTS

Fig. 1 denotes the position of the AF-linked somatic and germline mutations on a topographic composite model of human Cx40. Among all the reported AF-linked Cx40 mutants, the Q49X mutant is the only nonsense mutant, which leads to a premature truncation of the Cx40 polypeptide. This autosomal-dominant mutant was identified in seven AF patients in one Chinese family over generations (Yang et al., 2010a). The average age of the clinical identification of AF in Q49X carriers was 35 years old.

### The Q49X mutant failed to localize at cell-cell junctions

**Fig. 2:**
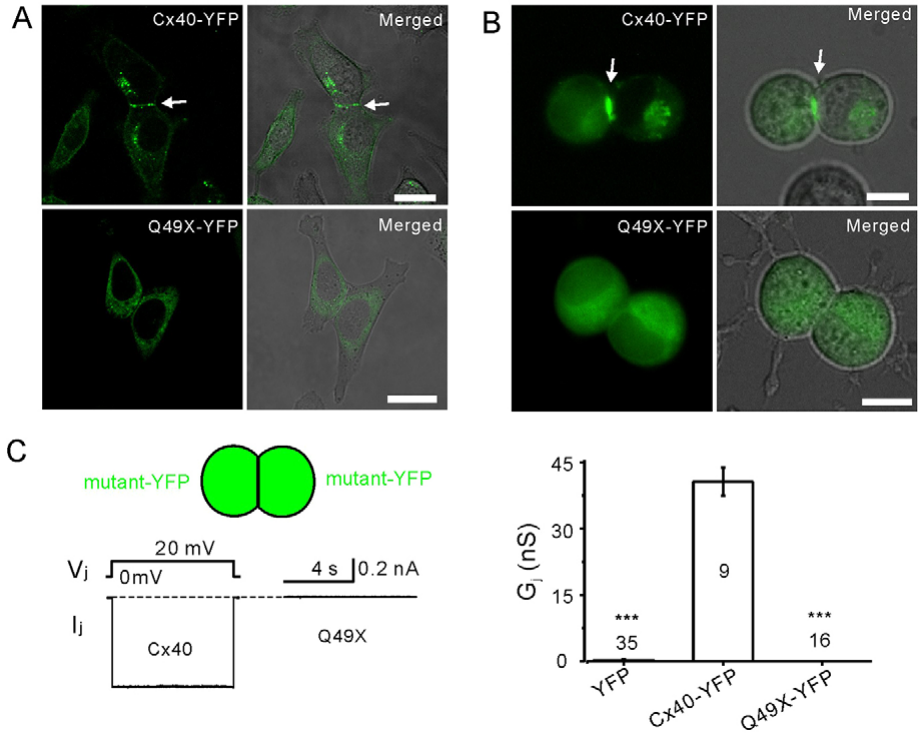
**The Q49X mutant failed to form morphological and functional gap junctions in cell pairs.** Confocal fluorescent images were presented separately or superimposed on phase-contrast images of HeLa cells (A) and N2A cells (B) expressing Cx40-YFP or Q49X-YFP. Wild-type Cx40 formed gap-junction plaque-like structures (arrows), but the Q49X mutant was retained in an intracellular compartment. Scale bars: 20 μm. (C) Junctional currents (I_j_) were consistently recorded in N2A cell pairs expressing Cx40, but I_j_ was not observed in cell pairs expressing Q49X. Bar graph summarizes G_j_s of N2A cell pairs expressing YFP (negative control), Cx40-YFP or Q49X-YFP. Numbers of cell pairs are indicated. Asterisks indicate a statistical significant difference (***P<0.001) from the G_j_ of Cx40-YFP.

YFP-tagged Cx40 and the Q49X mutant were expressed in HeLa cells to assess their ability to form gap-junction plaques at the cell-cell interfaces. In Cx40-expressing cells, there were 71% YFP-positive cell pairs exhibiting clear gap-junction plaque-like structures at cell-cell interfaces (*n*=330 cell pairs, Fig. 2A). The expression of untagged Cx40 when labeled with an anti-Cx40 C-terminal antibody exhibited a similar cell-cell interface localization in HeLa cells (data not shown), indicating that YFP-tagging at the C-terminus of Cx40 did not change the localization profile. In contrast, the Q49X mutant was found within intracellular compartments and failed to form gap-junction plaques at cell-cell interfaces (0%, *n*=300 cell pairs, Fig. 2A). Similarly, Cx40 was localized to cell-cell interfaces in N2A cells (Fig. 2B) and readily formed functional gap-junction channels (Fig. 2C). However, the Q49X mutant failed to display detectable gap-junction plaques in N2A cells (Fig. 2B) and the junctional current (I_j_) at 20 mV transjunctional voltage gradient (V_j_, Fig. 2C) was absent, indicating that the mutant failed to reach cell-cell interfaces and was therefore unable to form functional gap-junction channels. The macroscopic gap-junctional conductance (G_j_) of cell pairs expressing Cx40-YFP was similar to that of untagged Cx40 (data not shown), further suggesting that the YFP tag did not interfere with channel function.

### The Q49X mutant impairs the localization of co-expressed Cx40

**Fig. 3:**
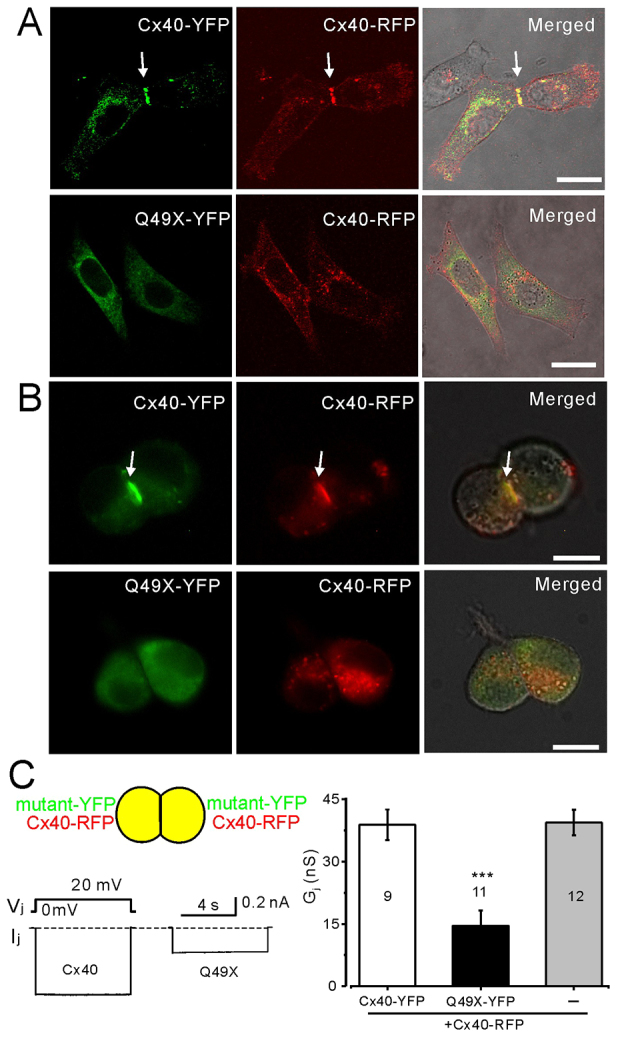
**Dominant-negative effects of Q49X on Cx40.** Confocal images of HeLa cell pairs (A) and N2A cells (B) are shown co-expressing Cx40-YFP and Cx40-RFP (top row) or Q49X-YFP and Cx40-RFP (bottom row). Cx40-YFP and Cx40-RFP were typically colocalized, forming putative gap-junction plaques (arrows). The Q49X mutant impaired the ability of Cx40 to form gap junctions. Scale bars: 20 μm. (C) Dual patch-clamp recordings of N2A cell pairs co-expressing Cx40 or mutant connexin with Cx40-RFP. Cell pair co-expressing Q49X and Cx40 showed a significant reduction in I_j_ compared with the cell pairs co-expressing Cx40-YFP and Cx40-RFP. Bar graph summarizes the junctional conductance of cell pairs co-expressing Cx40-YFP + Cx40-RFP, Q49X-YFP + Cx40-RFP (***P<0.001) or Cx40-RFP alone. Numbers of cell pairs are indicated.

To determine the effects of the mutant on Cx40 localization, we co-expressed Cx40 and the Q49X mutant in HeLa cells. As expected, Cx40-YFP and Cx40-RFP colocalized at cell-cell interfaces (65% cell pairs showed putative gap-junction plaques with both YFP and RFP, *n*=228 cell pairs) and, to a much lesser extent, to the same intracellular compartments (Fig. 3A). In cell pairs co-expressing Q49X and Cx40, no colocalization of Q49X-YFP and Cx40-RFP at the cell-cell interfaces was observed (0% from 278 cell pairs), and there was a great reduction in putative Cx40-RFP gap-junction plaques (4% cells showed gap-junction plaques from 278 cell pairs) at cell-cell interfaces (Fig. 3). Furthermore, only partial colocalization of Q49X and Cx40 was observed in intracellular compartments. Similar results were also observed in N2A cells (Fig. 3B). These results suggest that the Q49X mutant impaired Cx40 localization to cell membrane.

Functional tests on N2A cell pairs co-expressing Q49X and Cx40 demonstrated a substantial reduction in the G_j_ compared with the cell pairs expressing Cx40 (Fig. 3C). As a control, the G_j_ of cell pairs expressing Cx40-RFP alone was similar to that of cell pairs expressing both Cx40-YFP and Cx40-RFP (Fig. 3C). These results indicated that Q49X showed a dominant-negative effect on gap-junction function of Cx40.

### The Q49X mutant impairs the localization of Cx43

**Fig. 4:**
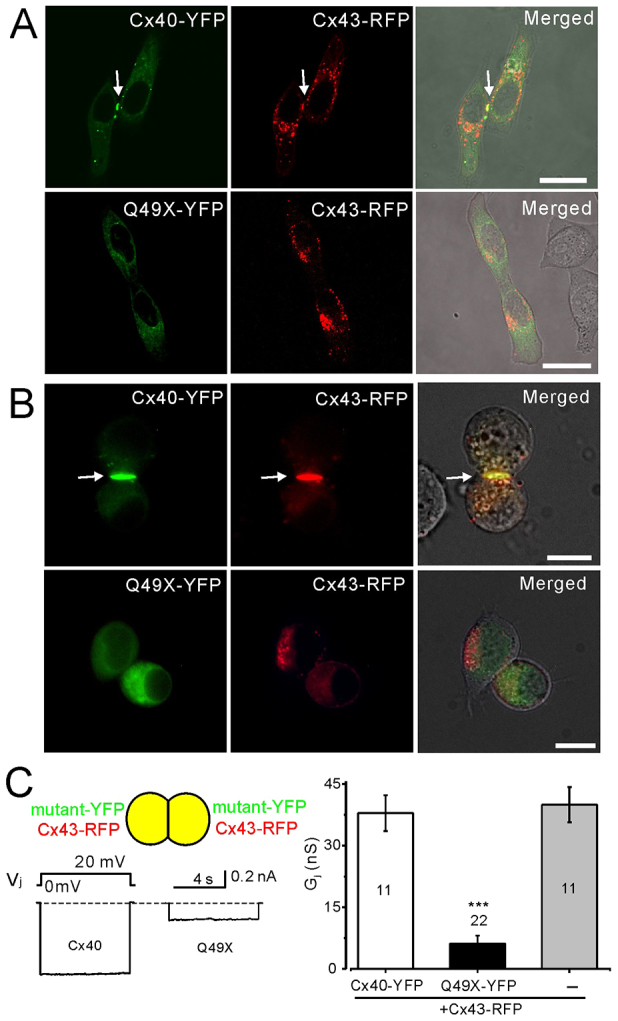
**Transdominant-negative effects of Q49X on Cx43.** Confocal fluorescent images of live cells were superimposed on phase-contrast images of HeLa (A) and N2A (B) cell pairs expressing fluorescent-protein-tagged connexins and/or the Q49X mutant. Putative gap-junction plaques were readily seen in cells co-expressing Cx40-YFP and Cx43-RFP (arrows), but not in cell pairs expressing mutant Q49X-YFP and Cx43-RFP. Scale bars: 20 μm. (C) Dual patch-clamp recordings of N2A cell pairs co-expressing Cx40 (or Q49X) with Cx43. Cell pairs co-expressing Q49X and Cx43 showed a significantly lower amplitude of I_j_ (and the calculated junctional conductance G_j_, ***P<0.001) compared with cell pairs co-expressing Cx40 and Cx43. Bar graph summarizes the junctional conductance for each mutant and connexin combination. Numbers of cell pairs are indicated.

Previous studies suggest that Cx40 and Cx43 can form heteromeric gap-junction channels (Valiunas et al., 2001; Lin et al., 2010). To identify whether the Q49X mutant affects the localization of Cx43, we co-expressed Q49X-YFP (or Cx40-YFP) and Cx43-RFP. As shown in Fig. 4A, co-expression of Cx40 and Cx43 resulted in their colocalization at sites of putative gap-junction plaques (Fig. 4A, 64% showed both YFP and RFP within the same gap-junction plaques, acquired from 211 cell pairs). However, co-expression of the Q49X mutant with Cx43 resulted in no putative gap-junction-plaque formation (0% from 245 cell pairs), with both YFP and RFP at the cell-cell interfaces, and a substantial reduction of Cx43-RFP gap-junction plaques (3% cells showed plaques from 245 cell pairs), indicating that the Q49X mutant reduced the ability of Cx43 from assembling into gap junctions at cell-cell interfaces. Similar results were also observed in N2A cells (Fig. 4B), suggesting that the Q49X mutant exhibited a transdominant-negative effect on the assembly of Cx43 into gap junctions at cell-cell interfaces.

As predicted from the negative effect of the mutant on the cell surface localization of Cx43, the G_j_ of the cell pairs co-expressing the mutant and Cx43 was significantly lower than that of co-expressing Cx40 and Cx43 (Fig. 4C), indicating a functional transdominant-negative effect of the Q49X mutant on Cx43. The G_j_ of cell pairs expressing Cx43-RFP alone was similar to that of cell pairs expressing both Cx40-YFP and Cx43-RFP (Fig. 4C), suggesting that the maximum amount of cell coupling had been reached.

### The Q49X mutant was retained in the ER

**Fig. 5:**
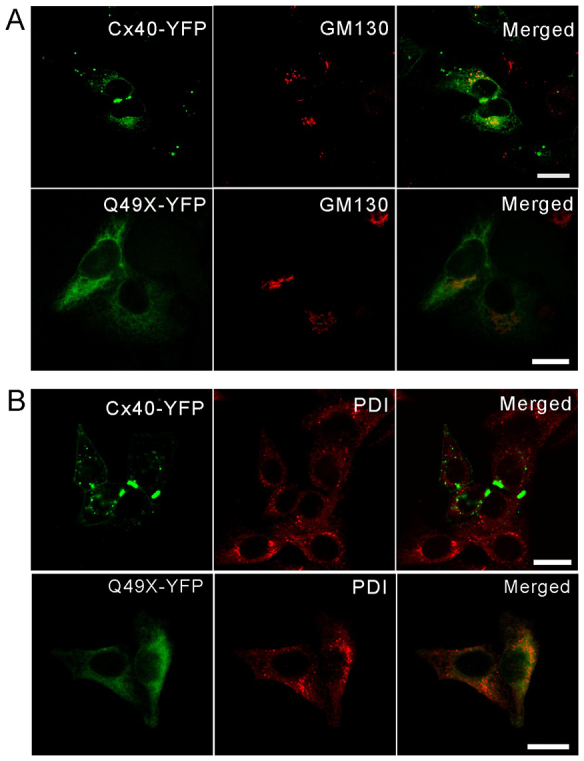
**Subcellular localization of the Q49X mutant in HeLa cells.** HeLa cells were engineered to express Cx40-YFP or Q49X-YFP prior to immunolabeling for a resident protein of the Golgi apparatus (GM130) (A) or the ER (PDI) (B). Note that the mutant primarily colocalized with PDI. Scale bars: 20 μm.

**Fig. 6:**
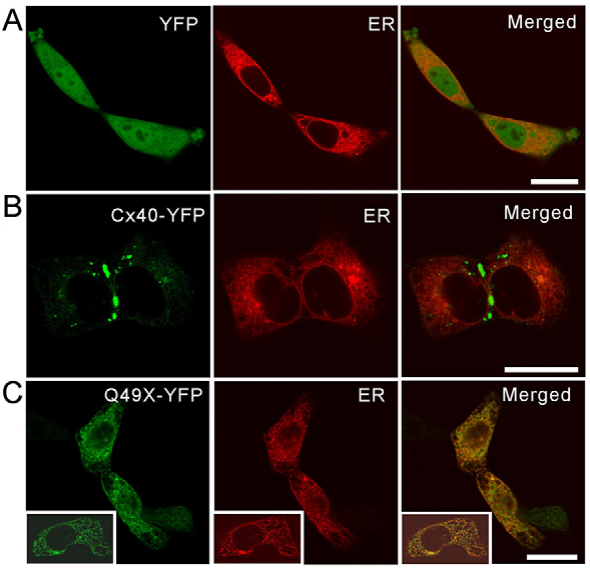
**The Q49X mutant colocalized with DsRed-ER in live HeLa cells.** HeLa cells were engineered to express YFP (A), Cx40-YFP (B) or Q49X-YFP (C) together with a red fluorescent marker of the ER (DsRed-ER). Note that the mutant colocalized extensively with DsRed-ER. Insets in C show another example of colocalization. Scale bars: 20 μm.

To determine the subcellular localizations of the mutant protein, Q49X-YFP-expressing HeLa cells were further immunolabeled with antibodies against either PDI, a resident protein of the ER, or GM130, a resident protein of the Golgi apparatus (Fig. 5). Q49X seemed to preferentially colocalize with PDI (Fig. 5B), indicating that the mutant acquires a steady-state residence in the ER. Partial colocalization of wild-type Cx40 (or Q49X) and GM130 was also observed (Fig. 5A). To further confirm the localization of the Q49X mutant to the ER, HeLa cells were engineered to co-express Q49X-YFP and DsRed-ER (live fluorescent marker for ER). Q49X shared a remarkably similar localization pattern with that of DsRed-ER (Pearson’s correlation=0.73±0.06, *n*=9, Fig. 6C). In contrast, Cx40 exhibited a much weaker colocalization with the ER (Pearson’s correlation=0.18±0.03, *n*=7, Fig. 6B). Unlike free YFP, the Q49X mutant was not localized to the nucleus (Fig. 6A,C). These findings suggest that the truncated Cx40 mutant Q49X was retained primarily within the ER.

### The untagged Q49X mutant impairs the function of co-expressed connexins

**Fig. 7:**
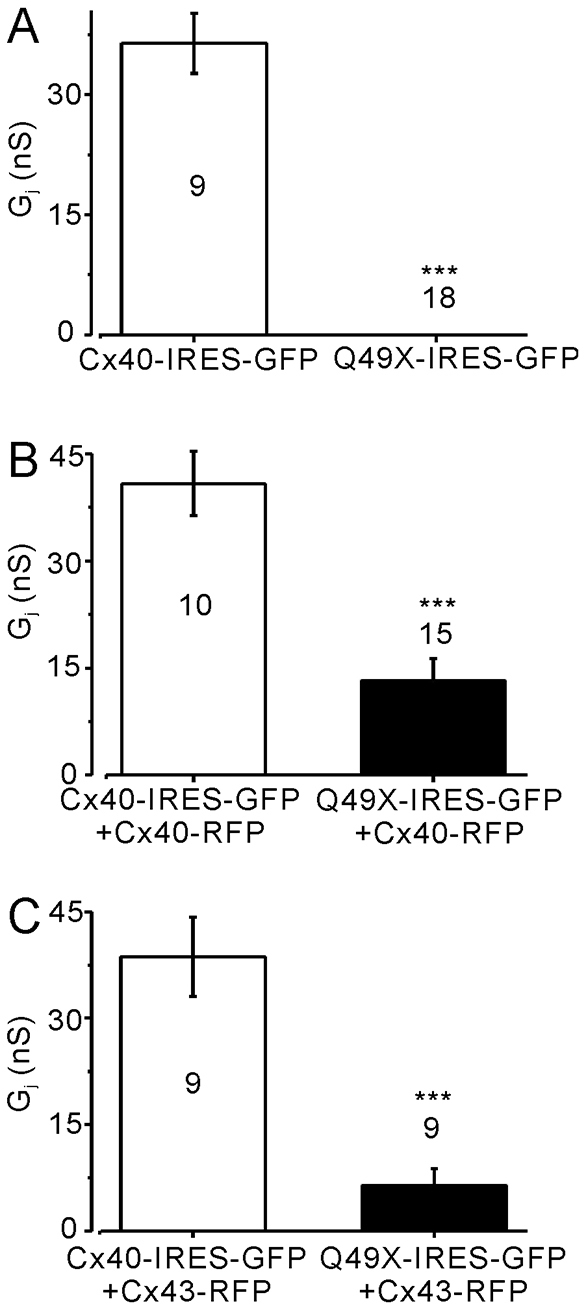
**Untagged Q49X failed to establish functional gap-junction coupling and dominantly inhibited the coupling of co-expressed Cx40 or Cx43 in N2A cells.** (A) N2A cell pairs expressing Q49X-IRES-GFP exhibited no coupling (G_j_=0 nS), unlike cell pairs expressing Cx40-IRES-GFP (***P<0.001). (B) Cell pairs co-expressing Q49X-IRES-GFP and Cx40-RFP exhibited significantly lower G_j_ (***P<0.001) compared with the cell pairs co-expressing Cx40-IRES-GFP and Cx40-RFP. (C) Cell pairs co-expressing Q49X-IRES-GFP and Cx43-RFP exhibited a significantly lower G_j_ (***P<0.001) than that obtained from the cell pairs co-expressing Cx40-IRES-GFP and Cx43-RFP. Number of cell pairs tested in each case is indicated.

To eliminate the possibility of the observed dominant-negative properties of the Q49X mutant was due to its YFP tag, the untagged Q49X mutant was co-expressed with fluorescent-protein-tagged Cx40 or Cx43. As shown in Fig. 7, cell pairs co-expressing the Q49X mutant and, via the bicistronic vector, GFP were completely uncoupled. Consistent with the results from the expression of the Q49X-YFP mutant, untagged Q49X also reduced the G_j_ when it was co-expressed with Cx40 or Cx43 (Fig. 7B,C). These results further support the finding that the loss-of-function Cx40 mutant was also dominant-negative to the function of both Cx40 and Cx43.

**Fig. 8:**
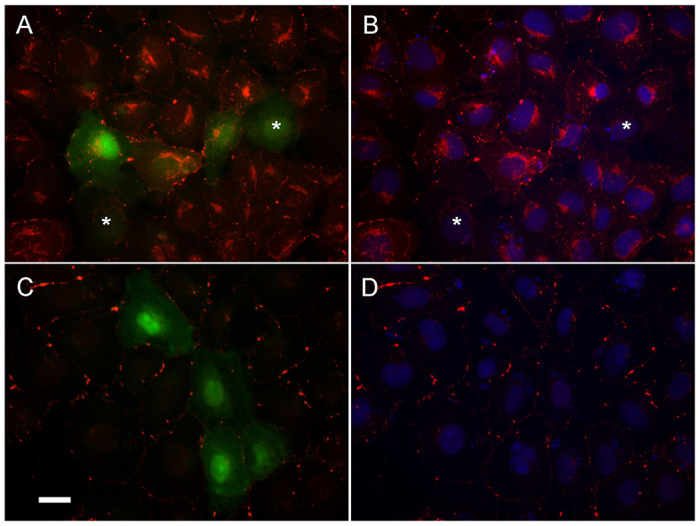
**Untagged Q49X mutant expression reduced the incidence of Cx43 gap-junction plaques in NRK cells.** NRK cells expressing the untagged Q49X mutant (green) were immunolabeled for Cx43 (red) before (A,B) and after (C,D) treatment with cycloheximide (30 μg/ml for 9 hours). The incidence of Cx43 gap-junction plaques between Q49X-expressing cells (green) was reduced in both conditions. B and D are from the same cells shown in A and C, respectively, but with Hoechst nuclei staining. Asterisks (A,B) indicate Q49X-expressing cells with an obvious reduction of intracellular Cx43. Scale bar: 20 μm.

To study whether the untagged Cx40 Q49X mutant is also able to alter the localization of endogenously expressed Cx43, we expressed the Q49X mutant in normal rat kidney (NRK) cells. Anti-Cx43 antibody labeling showed an abundant expression of Cx43 in the intracellular compartments and at the cell-cell interfaces in non-transfected NRK cells (Fig. 8A). Expression of the Q49X mutant (green cells in Fig. 8A) substantially reduced the apparent number of Cx43 gap-junction plaques at the interfaces between mutant-expressing cells (Fig. 8). Reduction of intracellular Cx43 was also observed in some cells expressing the mutant, especially in the perinuclear area (cells with asterisks in Fig. 8A,B). As expected, a 9-hour treatment of NRK cells with a protein-synthesis inhibitor, cycloheximide, substantially reduced the overall level of Cx43, especially in the intracellular compartments (Fig. 8C,D), yet Cx43 plaques were still prominent at the cell-cell interfaces. Similar to the effects of the Q49X mutant in the absence of cycloheximide, the mutant reduced the size and/or number of gap-junction plaques at the cell-cell interfaces between mutant-expressing cells in the presence of cycloheximide (Fig. 8C,D).

## DISCUSSION

Here, we characterized the first and only nonsense AF-linked germline Cx40 mutant. This mutation introduced a premature stop codon at amino acid position 49, predicting to generate a truncation mutant of Cx40, Q49X. This mutation is the most frequently found Cx40 mutant to date and affected individuals are prone to the onset of AF early in their adult life (see Table 1). Our findings in the mammalian cell lines clearly indicate that, if the Q49X mutant were expressed as a truncated protein, it would be unlikely to form morphological or functional gap junctions owing to its retention in the ER. Importantly, our data indicate that the mutant was potent at reducing the coupling conductance of co-expressed Cx40 and Cx43, suggesting that it exhibits both dominant- and transdominant-negative properties, respectively. The mechanism responsible for the dominant-negative properties of the mutant seems to be partially rooted in the ability of the mutant to sequester Cx40 and Cx43 in the ER, thereby reducing the ability of these connexins to reach the site to form functional gap junctions. Our results are consistent with a model in which impairment of the overall atrial gap-junction function plays an important role in promoting AF.

**Table 1:**
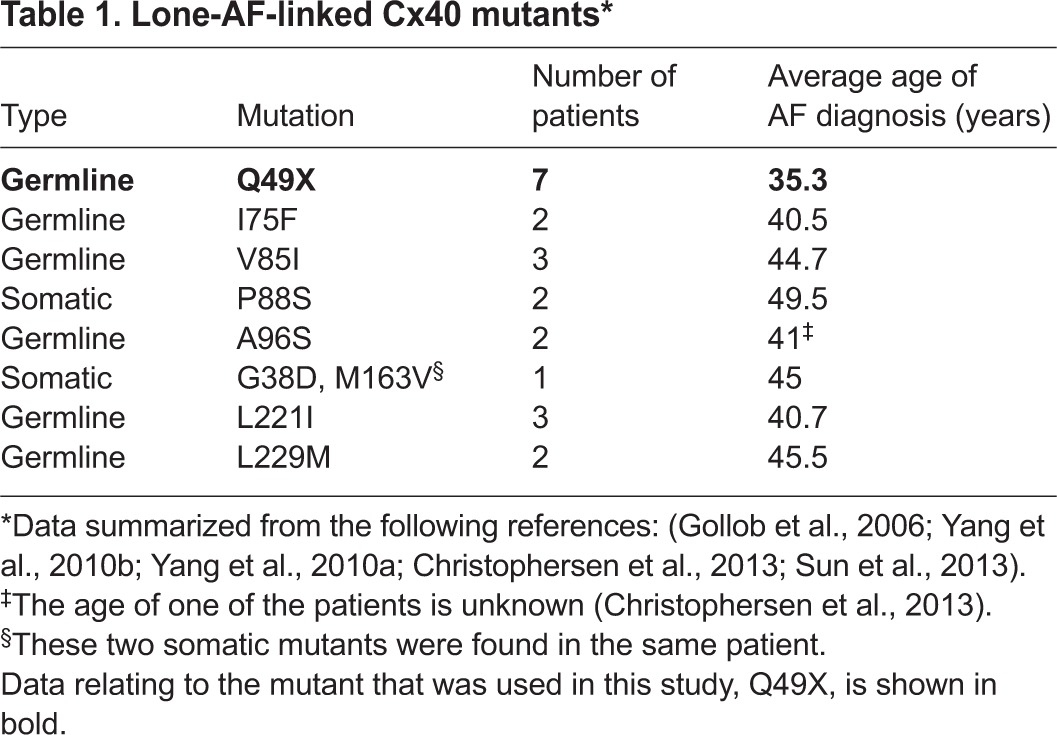
Lone-AF-linked Cx40 mutants*

AF is the most common cardiac arrhythmia and represents a major public health problem. Although AF has been traditionally regarded as a sporadic and acquired disease, further reports have suggested that genetic factors play an important role in the pathogenesis of AF (Fatkin et al., 2007; Xiao et al., 2011). The Framingham Heart Study indicated that the relative risk of AF in individuals with at least one parent with a history of AF is increased by 85% compared with people with no parental history of AF (Fox et al., 2004). Identification of specific genetic factors associated with AF, especially in lone AF cases, is of crucial need because these factors might provide mechanistic insights into the AF substrates and potential treatments. In addition to mutations in the genes encoding ion channels, calcium handling proteins, signaling molecules and structural proteins (Tsai et al., 2008; Xiao et al., 2011), recent genetic screenings identified somatic and germline mutations in the *GJA5* gene (encoding Cx40) associated with lone AF (Gollob et al., 2006; Yang et al., 2010b; Yang et al., 2010a). From these clinical cases, carriers of *GJA5* gene mutations had a particularly early onset of AF (Table 1). A somatic mutation in the gene encoding the other principal atrial connexin, Cx43, was also found in a lone AF patient (Thibodeau et al., 2010), indicating a possible important pathogenic role of atrial gap junctions in AF.

The molecular and cellular mechanisms by which Cx40 mutants cause AF is not well understood. Characterization of a few other Cx40 mutants linked to AF, such as G38D, I75F, P88S, A96S and L229M, strongly suggests that these mutants can affect the distribution and/or the function of gap junctions (Gollob et al., 2006; Sun et al., 2013). For example, when expressed in connexin-null cells, the G38D mutant exhibited reduced ability, and the P88S mutant failed, to form gap-junction plaques at cell-cell interfaces. Furthermore, the subcellular organelle localization of these mutants was not investigated nor was the effect of these mutants on the distribution of co-expressed wild-type Cx40 or Cx43 (Gollob et al., 2006). Other Cx40 mutants such as I75F, A96S and L229M exhibited no apparent defects in the ability to form gap-junction plaques (Gollob et al., 2006; Sun et al., 2013). Functionally, cell pairs expressing the mutants exhibited no (P88S and I75F) or very low levels of (A96S and G38D) functional coupling (Gollob et al., 2006; Sun et al., 2013). Furthermore, the mutants also substantially reduced the coupling of Cx40 (P88S, A96S and I75F) and Cx43 (P88S, A96S, I75F and L229M) gap junctions, suggesting that they exhibited dominant- and/or transdominant-negative properties on these atrial connexins (Gollob et al., 2006; Sun et al., 2013). However, the mechanism of action for how these mutants impaired overall gap-junction coupling seemed to be distinctly different from what we discovered in the current study. Here, we provide comprehensive experimental evidence that the Q49X mutant can be trapped in the ER as a truncated fragment of Cx40. Retention of the mutant in the ER likely resulted in its misfolding and targeting to the ER-associated degradation pathway where it would be eventually cleared from the cell (VanSlyke and Musil, 2002; Meusser et al., 2005). This might be the only AF-linked Cx40 mutant that fails the ‘quality control’ mechanisms in the ER, resulting in its retention in this organelle. It is interesting that the ER-retention of Q49X not only prevented the mutant from getting to cell-cell interfaces, but also greatly impaired co-expressed Cx40 and Cx43 from forming gap junctions in the reference cells used in this study. The mechanism of this dominant action of Q49X is not clear but we propose that it might interact with Cx40 and Cx43, causing these atrial connexins to be retained in the ER and ultimately be susceptible to ER-based degradation processes (VanSlyke and Musil, 2002; Meusser et al., 2005). This notion is supported by the following experimental evidence: (1) previous studies showed that Cx40 and Cx43 could co-oligomerize into homomeric or heteromeric channels (Gemel et al., 2006; Lin et al., 2010); thus, it is quite possible that the N-terminal fragment of Cx40 could indeed interact with Cx40 and Cx43; (2) the N-terminal domain of connexins has been reported to regulate the oligomerization process (Lagree et al., 2003); (3) our localization studies of cells co-expressing the Q49X mutant and Cx40 (or Cx43) resulted in a partial colocalization of Q49X and Cx40 (or Cx43) likely in the ER. The fact that the localization pattern of the mutant does not fully overlap with Cx40 or Cx43 when co-expressed suggests that the mutant might transiently interact with a population of the full-length connexin, allowing it to exit the ER and enter later secretory compartments, but this notion has yet to be established. Regardless, the presence of the mutant dramatically impairs the ability of Cx40 or Cx43 from forming functional gap junctions.

Because the mutation in *GJA5* introduced a premature translation termination codon at the Q49 position, the mutant might be subjected to nonsense-mediated mRNA decay (NMD) (Mendell et al., 2004; Rebbapragada and Lykke-Andersen, 2009). However, the coding region of the *GJA5* gene, encoding Cx40, is within a single exon (Dupays et al., 2003) without any exon-exon junctions for splicing, which reduces the possibility of the mutant having the exon-junction-complex-dependent NMD (Maquat, 2004). Although not all truncation mutations close to the initiation codon are subject to NMD (Perrin-Vidoz et al., 2002; Inácio et al., 2004), at present we cannot rule out NMD in Q49X patients owing to the inaccessibility to cardiac tissue from these patients, where the mutant would be expected to be expressed. If Q49X were subjected to NMD, then the overall abundance of Cx40 would be reduced, which could play a role in the pathogenesis of AF as suggested from genetic studies (Firouzi et al., 2004; Wirka et al., 2011). However, a 50% complement of Cx40 might not evoke a pathology in humans, opening up the possibility that the mutant allele is contributing to AF. Future studies in which postmortem or biopsy material can be obtained from Q49X patients will be necessary to resolve this outstanding question.

It has been known for some time that there was a link between Cx40-mediated gap-junction coupling and arrhythmias. For instance, mice lacking the *Cx40* gene exhibit a decrease in the action-potential conduction velocity (Kirchhoff et al., 1998; Simon et al., 1998) and an increased vulnerability to inducible arrhythmias (Kirchhoff et al., 1998; Hagendorff et al., 1999). More recent studies indicate that Cx40 plays a role in increasing the heterogeneity in the conduction velocity in atrial appendages (Bagwe et al., 2005; Leaf et al., 2008). However, an increase in the conduction velocity was found in cultured strands of atrial myocytes obtained from Cx40 knockout mice (Beauchamp et al., 2006), raising concerns as to the overall relationship between Cx40 and atrial arrhythmias in genetically modified mouse models. These inconsistencies have prompted the need to consider human genetic studies to further assess the role of Cx40 in AF (Jansen et al., 2010). To that end, Cx40 gap-junction expression and remodeling have been documented in both animal models and in the atrial tissues of individuals with AF (Kostin et al., 2002; Nattel et al., 2007; Duffy and Wit, 2008; Severs et al., 2008). Sporadic somatic mutations were identified in the *Cx40* gene in lone AF patients, leading to a genetic mosaicism in the atrial tissues (Gollob et al., 2006). In this case, clusters of mutant-expressing cells border with non-mutant-expressing cells in the atria, leading to regional heterogeneity in coupling. It is not clear whether the somatic mutant-expressing atrial myocytes also express wild-type Cx40 to allow the formation of Cx40-mutant and wild-type heteromeric gap junctions. Recently, autosomal-dominant germline Cx40 mutants were found to be linked to early-onset lone AF (Yang et al., 2010b; Yang et al., 2010a; Sun et al., 2013). All of the lone-AF-linked germline Cx40 mutants co-segregate with AF in the families up to three generations (Yang et al., 2010b; Yang et al., 2010a; Sun et al., 2013). This association was the most pronounced for the Q49X mutant, which was found in seven family members spread over generations. Our current study indicated that this loss-of-function mutant is capable of exhibiting dominant and transdominant actions on Cx40 and Cx43, respectively, providing experimental evidence for a possible role of the Cx40 mutant in causing an impaired atrial gap-junction function. It is worth noting that germline Cx40 mutants are expected to be expressed in all atrial myocytes and not confined to regions of the myocardium as is the case for somatic mutants. Depending on the pre-existing regional heterogeneity of the expression levels of Cx40 and Cx43 (Vozzi et al., 1999; Kanagaratnam et al., 2002; Kostin et al., 2002), the germline Cx40 mutants might cause different levels of impairments in various regions of the atrial tissues and increase the heterogeneity of the action-potential conduction velocity (Kleber et al., 2002; Spach et al., 2007), which might promote re-entrant arrhythmias. Finally, because it is well-known that AF prevalence increases sharply with aging, other co-morbidities might also contribute to the manifestation of the disease. Factors associated with the aging process include reduction and/or redistribution of ion channels and gap junctions (Kostin et al., 2002; Spach et al., 2007), and increased fibrosis (Stein et al., 2008), all of which could increase the heterogeneity of the atrial tissue and clinical presentation of the disease.

In summary, mutations in the *Cx40* gene, especially those with dominant and transdominant properties on the function of endogenous connexins, are linked to a much earlier onset of AF than the general population, indicating a crucial role of atrial gap junctions in the pathogenesis of AF. The current study is focused on model cells in which either no background gap junctions (HeLa and N2A cells) or only Cx43 gap junctions (NRK cells) are expressed. These mammalian model cells are excellent in delineating individual actions of a mutant and its interactions with a particular connexin. Future studies are required to examine whether the mutant also has similar actions in the native human or animal atrial myocytes.

## MATERIALS AND METHODS

### Plasmid construction

Human wild-type Cx40-YFP, Cx40-RFP, Cx43-RFP and Cx40-IRES-GFP expression vectors were generated as described earlier (Sun et al., 2013). Mutant Cx40 Q49X-YFP was generated by NorClone Biotech Laboratories (London, ON, Canada). DNA sequencing confirmed that the sequence encoding the first 48 amino acids of human Cx40 was directly fused in frame to YFP (Q49X-YFP). Untagged Q49X-IRES-GFP was generated from Cx40-IRES-GFP by using the QuikChange site-directed mutagenesis kit (Stratagene, La Jolla, CA) with the following primers: forward 5′-TGGGGGGATGAGTAGGCTGATTTCC-3′, reverse 5′-GGAAATCAGC -CTACTCATCCCCCCA-3′. All connexin clones were sequenced to confirm the fidelity of the nucleotide sequence.

### Connexin localization studies

Yellow fluorescent protein (YFP)-tagged wild-type and mutant connexins were localized following expression in HeLa or N2A cells. These cell lines are gap-junction-deficient, easily transfected with connexin cDNA constructs and capable of assembling the expressed connexin into morphological and functional gap junctions. Briefly, cells were grown to 70–80% confluence in glass-bottom dishes (MaTek, Ashland, MA) at 37°C in Dulbecco’s modified Eagle’s medium (DMEM) supplemented with 10% fetal bovine serum (FBS). X-tremeGENE HP DNA transfection reagent (Roche Applied Science, Indianapolis, IN) was used to transfect cDNAs encoding mutant or wild-type connexins. To mimic the patient context, in which both wild type and the mutant are co-expressed in the same cells, cDNAs encoding the mutant and Cx40 (or Cx43) were co-transfected at 1:1 ratio. Live HeLa cells were visualized with a confocal microscope (Zeiss LSM 510 META, Jena, Germany) or a CCD camera (Media Cybernetics, Rockville, MD) mounted on a fluorescent microscope (Olympus BX51) using a 40× water-immersion lens.

For subcellular localization of mutant connexin proteins, HeLa cells were rinsed in PBS (phosphate buffered saline) 24 hours after transfection. The cells were fixed for 10 minutes in 1:1 acetone/methanol at −20°C and blocked for 1 hour with 5% BSA in PBS. Antibodies against the resident proteins of the ER [protein disulfide isomerase (PDI), Stressgen Biotechnologies] or Golgi apparatus (GM130, BD Biosciences) were incubated for 1 hour at room temperature. Cells were washed twice and subsequently labeled for 30 minutes with Alexa-Fluor-594-conjugated secondary antibody. Following further washing, the coverslips were mounted on glass slides in preparation for confocal microscopy. For live-cell studies, cells were co-transfected with YFP-tagged mutant (or Cx40) and a DsRed-tagged ER marker protein (DsRed-ER, Clontech, Mountain View, CA). Double-transfected cells were observed with a confocal microscope 24 hours later. Pearson’s correlation coefficient was measured with Image Pro-Plus software (Media Cybernetics, Rockville, MD) to estimate the colocalization of the mutant and the organelle markers. Pearson’s correlation coefficient=1 represents perfect colocalization and zero represents random localizations.

NRK cells (American Type Culture Collection, Manassas, VA) were cultured in DMEM supplemented with 10% FBS. NRK cells were transfected with cDNA vector Cx40 Q49X-IRES-GFP. 24 hours after transfection, cycloheximide (30 μg/ml) was added in some dishes for 9 hours in 37°C incubator to block protein synthesis. Both cycloheximide- treated and untreated cells were fixed with 4% paraformaldehyde for 10 minutes at room temperature. The cells were permeabilized for 10 minutes with 0.2% Triton X-100 in PBS. After two washes, the cells were blocked with 3% BSA and then labeled overnight at 4°C with rabbit anti-Cx43 antibody (1/300 dilution in 2% BSA/PBS, Sigma, St Louis, MO). The secondary antibody was conjugated with Alexa Fluor 594 (Invitrogen). Counterstaining with Hoechst 33342 (10 μg/ml) for 2 minutes was used to show cell nuclei. Fluorescent images were obtained from a CCD camera (Media Cybernetics, Rockville, MD) mounted on a fluorescent microscope (Olympus BX51) using a 40× water-immersion lens.

### Electrophysiological studies

Electrophysiological recordings were carried out in the connexin-deficient N2A cells engineered to express wild-type or mutant connexins. To test the effect of the Q49X-YFP mutant on the function of Cx40-RFP (or Cx43-RFP), we co-transfected these two constructs at 1:1 ratio. The dual whole-cell patch-clamp technique was performed to assess the gap-junctional conductance between cell pairs co-expressing mutant and wild-type connexins (Gollob et al., 2006; Xin et al., 2010). Briefly, the junctional current was recorded via an amplifier (Axopatch 700A, Molecular Devices, Sunnyvale, CA) and the analog signals were digitized at a sampling rate of 10 kHz with an analog-to-digital converter, Digidata 1322A (Molecular Devices, Sunnyvale, CA), and analyzed with pClamp9 software. Each cell of a pair was initially voltage-clamped at a common holding potential of 0 mV. To evaluate coupling conductance, 20-mV pulses for 7 seconds were applied to one cell to establish a transjunctional voltage gradient (V_j_), while the junctional currents (I_j_) were measured in the other cell. Macroscopic gap junctional conductance (G_j_) was calculated as follows: G_j_=I_j_/V_j_. Data were obtained from multiple independent transfections. To reduce possible cytoplasmic bridge contamination on the G_j_ measurement, on occasion we included the gap-junction-impermeable high-molecular-weight dextran Texas red dye (0.25 mg/ml, MW=10,000 Da) in the pipette solution in one cell of the pair. Only cell pairs that did not show transfer of the dextran were selected for G_j_ analysis. In some experiments, we used the gap-junction blockers carbenoxolone (200–400 μM) or flufenamic acid (50 μM) to ensure that the measured G_j_ was due to gap-junction channels.

### Data analysis

All data are expressed as mean ± s.e.m. The statistical comparison of multiple groups of data was performed using one-way ANOVA with Student-Newman-Keuls test with SPSS software. An unpaired Student’s *t-*test was used to test statistical significance if data were from two groups. Statistical probability of *P*<0.05 was considered significant (***P<0.001 shown in figures).
